# Association of the Reproductive Period with Decreased Estimated Glomerular Filtration Rate in Menopausal Women: A Study from the Shanghai Suburban Adult Cohort and Biobank (2016–2020)

**DOI:** 10.3390/ijerph181910451

**Published:** 2021-10-05

**Authors:** Yuting Yu, Qi Zhao, Yonggen Jiang, Na Wang, Xing Liu, Yun Qiu, Junjie Zhu, Xin Tong, Shuheng Cui, Maryam Zaid, Jing Li, Jianguo Yu, Genming Zhao

**Affiliations:** 1 Key Laboratory of Public Health Safety of Ministry of Education, School of Public Health, Fudan University, Shanghai 200032, China; 19111020017@fudan.edu.cn (Y.Y.); zhaoqi@shmu.edu.cn (Q.Z.); na.wang@fudan.edu.cn (N.W.); liuxing@fudan.edu.cn (X.L.); qiuyun2018@fudan.edu.cn (Y.Q.); zhujunjie233@163.com (J.Z.); danmoweiliangtx@gmail.com (X.T.); cuishuheng1995@outlook.com (S.C.); maryzaid@fudan.edu.cn (M.Z.); 2 Songjiang District Center for Disease Control and Prevention, Shanghai 201600, China; sjjkbgs@163.com; 3 Department of Preventive Medicine, Zhongshan Community Health Center, Shanghai 201613, China; zhongshanlijing@163.com; 4 Zhongshan Community Health Center, Shanghai 201613, China

**Keywords:** reproductive period, chronic kidney disease, menopausal, glomerular filtration rate

## Abstract

In previous studies, it has been documented that a short reproductive period is associated with a higher risk of diabetes, cardiovascular disease, and chronic kidney disease. This study aims to investigate the association of the reproductive period length with decreased renal function. This study obtained data from “the Shanghai Suburban Adult Cohort and Biobank”. An estimated glomerular filtration rate (eGFR) below 60 mL/min/1.73 m^2^ indicated decreased renal function during follow-up. Participants were grouped into quintiles by reproductive period. Logistic regression analysis was performed to examine the association between the reproductive period and decreased renal function. A total of 5503 menopausal women with baseline eGFR > 60 mL/min/1.73 m^2^ were included. Age, eGFR, and metabolic equivalent of task (MET) at baseline were 61.0 (range, 36.0–74.0) years, 92.2 (range, 60.1–194.5) mL/min/1.73 m^2^, and 1386 (range, 160–6678), respectively. A reproductive period of 37–45 years was associated with a lower risk of decreased eGFR (OR: 0.59, 95% CI: 0.35–1.00, *p* = 0.049) after adjusting for confounding variables. METs decreased the risk of decreased eGFR in women with a reproductive period of 37–45 years (OR: 0.43, 95% CI: 0.23–0.81, *p* = 0.010). Women with a longer reproductive period have a lower risk of decreased renal function. METs had an opposite influence on renal function in women with longer (decreased risk) or shorter (increased risk) reproductive periods.

## 1. Introduction

Chronic kidney disease (CKD) is a progressive and lifelong disease causing a severe public health problem [1]. CKD is characterized by abnormalities of kidney structure or function that are present for >3 months, including a glomerular filtration rate (GFR) < 60 mL/minute/1.73 m^2^ or other evidence of kidney damage, such as albuminuria or abnormal kidney structure detected by imaging [1]. There were 752.7 million CKD patients worldwide in 2016, of which 55% were women [2]. CKD complications can include cardiovascular disease (CVD), hypertension, anemia, bone disease, electrolyte abnormalities, and, in the end stages, uremia [1,3]. CKD is a risk factor for mortality [4,5,6]. Compared with men, women probably benefit from the effects of estrogen on the renin–angiotensin–aldosterone system before menopause, but menopause increases the risk of CVD and osteoporosis, and most women with CKD (including those with dialysis requirements) are menopausal [7,8].

The female reproductive period, commonly regarded as between menarche and menopause, correlates with endogenous estrogen exposure in clinical studies [9,10,11,12]. The positive relationship between reproductive period and risk of CKD has been primarily revealed [13,14,15]. A recent study has shown that a shorter reproductive period and an older menarche age had significant associations with CKD [16], but the underlying mechanism needs elucidation. Studies showed that reproductive factors such as reproductive period, age at menarche, and age at menopause had significant associations with diabetes, metabolic syndrome, and CVD [17,18,19], increasing the risk of CKD. For example, one study showed a negative correlation between the reproductive period and the Framingham score [20]. Another study showed that a longer reproductive period was significantly associated with a lower prevalence of metabolic syndrome [21]. 

The metabolic equivalent of task (MET) concept represents a simple, practical, and easily understood procedure for expressing the energy cost of physical activities as a multiple of the resting metabolic rate [22,23]. MET of Chinese women is generally high [24,25]. Recent studies showed protective effects of physical activity and MET on osteoporosis [26,27,28] and CVD [29,30,31] in menopausal women. However, there is a lack of studies assessing physical activity on eGFR among women with different reproductive periods.

Therefore, this study aimed to investigate the association of the length of the reproductive period with decreased estimated GFR (eGFR) and whether performing physical activity can help reduce the risk of decreased eGFR.

## 2. Materials and Methods 

### 2.1. Study Design and Patients

This study was based on the data obtained from the “Shanghai Suburban Adult Cohort and Biobank (SSACB)”, which is a community-based cohort study conducted by the School of Public Health of Fudan University in collaboration with the Shanghai Songjiang Center for Disease Prevention and Control from June 2016 to August 2020 [32]. The SSACB study used a representative sample living in a suburban area with rapid urbanization, seven communities of Songjiang and Jiading, and a local population-based information system. Relevant information about the SSACB was previously described [32]. The baseline was performed between June 2016 and December 2017, and the first follow-up was performed between June 2019 and August 2020.

A multistage, stratified, clustered sampling was used to recruit the study participants. The inclusion criteria were (1) 20–74 years of age, and (2) natives of Shanghai municipality or have lived in Shanghai for at least 5 years. The exclusion criteria were (1) unable to consent, (2) pregnancy, (3) critical illness such as cancer, stroke, cirrhosis, cardiorespiratory failure, and hyper- or hypothyroidism, or (4) history of organ transplant or on dialysis treatment. For the present study, 5627 menopausal women with non-decreased eGFR status at baseline were included. After excluding women with incomplete data (physical examination, questionnaire survey, or laboratory measurement), 5503 eligible subjects were finally included in the longitudinal analysis.

The study protocol was approved by the ethical review committee of the School of Public Health of Fudan University (IRB approval number 2016-04-0586). Written informed consent has been provided by all participants.

### 2.2. Data Collection and Laboratory Measurements

The date of birth, marital status, education level, smoking status, alcohol consumption, physical activity, self-reported medical history of chronic diseases, and pregnancies were assessed using a structured questionnaire administered by trained interviewers. The education level was defined as low (primary education or below), intermediate (secondary general or vocational education), or high (university or college). Smoking status was defined as smoking more than one cigarette per day for six months. Smoking status was classified as never, former, or current. Alcohol consumption status was recorded as yes or no. Height and weight were measured in duplicates based on standardized protocols, and average values were determined. The body mass index (BMI) (kilograms per square meter) was defined as the weight in kilograms divided by the square of height in meters (kg/m^2^). BMI was assessed as a categorical variable (≤23.9, 24–27.9, ≥28) [33]. Patient self-reported data were validated using ICD-10 codes in matched medical data.

Participants were required to fast overnight for >8 h to obtain their fasting venous blood specimens the next morning. Their blood samples were used to perform blood biochemical assessments and routine blood tests in the DiAn Medical Laboratory Center. Serum creatinine (Scr) levels were measured using enzymatic methods on a Roche C702 automatic biochemical analyzer [32,34,35].

### 2.3. Diabetes, Hypertension, and Hyperlipidemia in Menopausal Women

In this study, menopause was defined as a retrospectively diagnosed cessation of menstruation for >12 months. The reproductive period was defined as the age at menopause (years, continuous variable) minus the age at menarche (years, continuous variable). The reproductive period was then assessed as a quintile categorical variable (18–31, 31–33, 33–35, 35–37, 37–45 years) in the analyses.

The diagnosis of T2DM was defined according to the International Diabetes Federation criteria of fasting plasma glucose (FPG) level ≥ 7.0 mmol/L, or HbA1c ≥6.5% or a previous diagnosis of T2DM [36]. Hypertension was defined as systolic blood pressure (SBP) ≥ 140 mmHg and/or diastolic blood pressure (DBP) ≥ 90 mmHg and/or a previous diagnosis of hypertension. Hyperlipidemia was defined as total cholesterol (TC) ≥ 6.20 mmol/L or triglycerides (TG) ≥ 2.30 mmol/L, HDL-C < 1.00 mmol/L, LDL-C ≥ 4.10 mmol/L, and/or a previous diagnosis of hyperlipidemia.

### 2.4. Metabolic Equivalents of Tasks

MET levels were obtained from the 2000 Compendium of Physical Activities to include moderate-intensity activities between 3 and 6 METs and vigorous-intensity activities as >6 METs [37]. The weighted MET-minutes per week (MET × min/w) were calculated as duration × frequency per week × MET intensity, which were summed across activity domains to produce a weighted estimate of total physical activity from all reported activities per week (MET × min/w). MET was converted to the logarithm of MET (logMET) for statistical analysis.

### 2.5. Decreased Renal Function

The eGFR was calculated to estimate the kidney function using the Chronic Kidney Disease Epidemiology Collaboration (CKD-EPI) equation for the Chinese population [38]:

(1) $$\mathrm{eGFR}\;\left(\mathrm{mL}∕\min∕1.73{\;m}^{2}\right)=141\times \min{\left(\mathrm{Scr}∕\kappa ,1\right)}^{\alpha }\times \max{\left(\mathrm{Scr}∕\kappa ,1\right)}^{-1.209}\times 0.993^{\mathrm{Age}}\times 1.018\left[\mathrm{if}\;\mathrm{female}\right]$$

where Scr is the serum creatinine (mg/dL), κis 0.7 for females, α is −0.329 for females, min indicates the minimum of Scr/κ or 1, and max indicates the maximum of Scr/κ or 1.

Decreased renal function was defined as an eGFR value below 60 mL/min/1.73 m^2^, according to the KDIGO criteria [1]. The participants were divided into four categories according to the CKD classification by the National Kidney Foundation [1]: normal eGFR, ≥90 mL/min/1.73 m^2^; mildly decreased eGFR, 60–89 mL/min/1.73 m^2^; moderately decreased eGFR, 30–59 mL/min/1.73 m^2^; and severely decreased eGFR, 15–29 mL/min/1.73 m^2^.

### 2.6. Follow-Up

The cohort follow-ups consisted of in-person surveys and annual checks of the local health information system. The first follow-up was conducted to update the longitudinal data on measured demographic characteristics and laboratory data, including serum creatinine, and to collect the new data on exposures to different risk factors. 

### 2.7. Statistical Analysis

The data were analyzed using Stata 16.0 (StataCorp, Dallas, TX, USA). All the findings were based on complex sample data and presented as weighted results. Continuous data were expressed as median (range) and analyzed using the Kruskal–Wallis rank test. The Pearson chi-square test was used for categorical data presented as *n* (%). Logistic regression analyses were conducted to determine the odds ratios (ORs) and 95% confidence intervals (CIs) to estimate the association between the reproductive period and decreased eGFR. Multivariable models were constructed for adjusting for confounding factors according to the previous literature [9,12,13,19,39]. Model 1: adjust for age; model 2, adjust for age, BMI, parental history of chronic kidney disease, age at menarche, pregnancies, and hormone replacement therapy; model 3, adjust for age, BMI, parental history of chronic kidney disease, age at menarche, pregnancies, hormone replacement therapy, education, smoking, alcohol intake, hypertension, hyperlipidemia, blood urea nitrogen, hemoglobin, albumin, uric acid, and metabolic equivalents. The restricted cubic spline model was used to evaluate the relationship between the reproductive period and decreased eGFR. The reference point of the reproductive period was 18 years, which was the lower bound of the reproductive period. The marginal analysis of the MarginsContPlot (MCP) command was used to assess the marginal effect of physical activity on fixed reproductive period on decreased eGFR. *p*-values < 0.05 were considered statistically significant.

## 3. Results

### 3.1. Characteristics of the Participants

A total of 5503 menopausal women were included in this prospective cohort. The baseline participants’ characteristics according to the length of the reproductive period are summarized in Table 1. The median age of all subjects was 61.0 (range, 36.0–74.0) years, the median BMI was 24.4 (range, 14.6–43.6) kg/m^2^, and the median reproductive period was 34.0 (range, 18.0–45.0) years. As shown in Table 1, with the increase in reproductive period, age decreased (*p* < 0.001), age at menarche was younger (*p* < 0.001), age at menopause was older (*p* < 0.001), the proportion of only one child was higher (*p* < 0.001), BMI was higher (*p* < 0.001), and eGFR was higher (*p* < 0.001). Comorbid conditions such as hypertension (*p* = 0.15) and Type 2 diabetes mellitus (*p* = 0.85) were not statistically significantly different among quintiles. METs were not different among the reproductive period quintiles.

### 3.2. Association between the Reproductive Period and Decreased Renal Function

The total incidence of decreased eGFR was 3.5%. The proportion of menopausal women with decreased eGFR in the reproductive period quintiles were 5.7% (63/1103), 3.7% (41/1105), 2.6% (28/1098), 2.7% (31/1102), and 2.7% (24/1095), respectively. Women with a shorter reproductive period exhibited a higher incidence of decreased eGFR compared with other women (*p* < 0.001) (Supplement Table S1).

### 3.3. Multivariable Analyses for the Development of Decreased Renal Function

**Figure 1:**
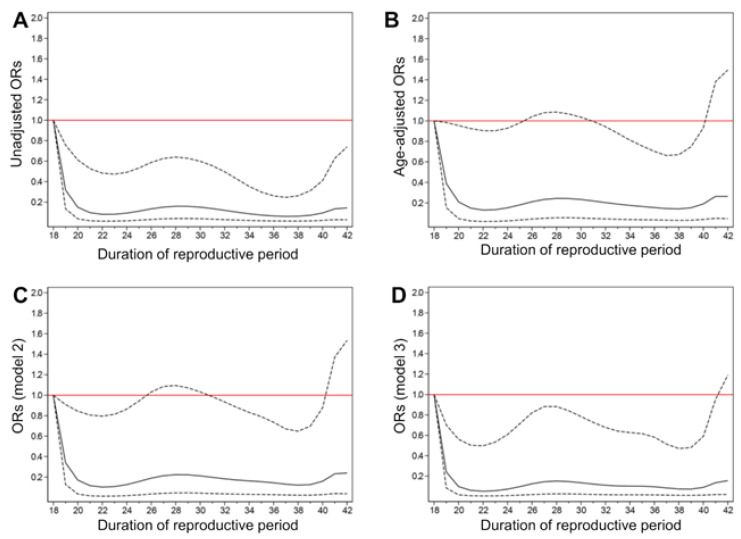
Odds ratios (ORs) (solid line) and 95% confidence intervals (dashed lines) of the reproductive period for estimated glomerular filtration rate (eGFR) < 60 mL/min/1.73 m^2^ (reference: 18 years). (**A**) Unadjusted ORs; (**B**) Model 1: adjust for age; (**C**) Model 2: adjust for age, body mass index (BMI), parental history of chronic kidney disease, age at menarche, pregnancies, and hormone replacement therapy; (**D**) model 3: adjust for age, BMI, parental history of chronic kidney disease, age at menarche, pregnancies, hormone replacement therapy, education, smoking, alcohol intake, hypertension, hyperlipidemia, blood urea nitrogen, hemoglobin, albumin, uric acid, and metabolic equivalent.

The median follow-up was 36 months. Table 2 and Figure 1 show the ORs (95% CIs) for developing eGFR < 60 mL/min/1.73 m^2^ according to the reproductive period. Compared with women with a reproductive period of 18–31 years, the ORs of decreased eGFR were significantly different in those with a reproductive period of 37–45 years to other reproductive period quintiles in models 1 (OR = 0.53, 95% CI: 0.30–0.96, *p* = 0.036) and 3 (OR = 0.59, 95% CI: 0.35–1.00, *p* = 0.049). Every 1-year increase in the length of the reproductive period was associated with a 4% lower rate of developing eGFR < 60 mL/min/1.73 m^2^ (OR = 0.96, 95% CI: 0.93–1.00, *p* = 0.030) in model 1 (Table 2). The relationship between the length of the reproductive period and decreased eGFR risk is depicted in the restricted cubic spline curves in Figure 1.

### 3.4. Interaction between Reproductive Period and Physical Activity on Decreased Renal Function

**Figure 2:**
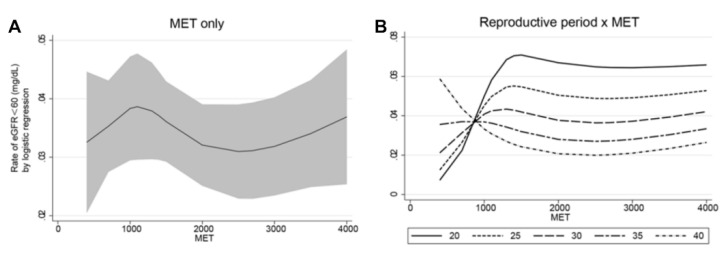
The marginal effect of metabolic equivalents in women with different fixed reproductive periods on decreased estimated glomerular filtration rate (eGFR). (**A**) MET on developing eGFR < 60 mL/min/1.73 m^2^. (**B**) The interaction effect of the length of the reproductive period and amount of physical activity on developing eGFR < 60 mL/min/1.73 m^2^. Adjusted variables: age, body mass index, parental history of chronic kidney disease, age at menarche, pregnancies, hormone replacement therapy, education, smoking, alcohol intake, hypertension, hyperlipidemia, blood urea nitrogen, hemoglobin, albumin, and uric acid.

Table 3 and Figure 2 show the interaction effect of the length of the reproductive period and the amount of physical activity on developing eGFR < 60 mL/min/1.73 m^2^. After adjusting for demographic, metabolic, and reproductive factors, physical activity (quantified by MET) reduced the risk of developing eGFR < 60 mL/min/1.73 m^2^ in those with a reproductive period of 37–45 years (*p* = 0.010). The effect of physical activity on decreased eGFR by different lengths of the reproductive period is depicted in Figure 2: the log of the odds of developing eGFR < 60 mL/min/1.73 m^2^ decreases with METs in women with a reproductive period of 35–40 years until 2500 METs, while it increases sharply with METs in women with a reproductive period of 20–30 years.

## 4. Discussion

This study aimed to investigate the association of the length of the reproductive period with decreased eGFR and the effect of physical activity on the risk of decreased renal function. The results suggest that women with a longer reproductive period might have a lower risk of developing decreased renal function. Performing physical activity could reduce the risk of decreased renal function in women with a longer reproductive period, while it tended to increase the risk of decreased eGFR in women with a shorter reproductive period.

A previous study reported the association between the reproductive period and CKD; after adjustment for confounders, women with a shorter reproductive period had a higher risk of developing CKD [16]. Kang et al. [15] also showed that women with longer reproductive periods had a lower risk of CKD. These two studies support the present study. Although none of the studies examined the possible mechanisms, the results might suggest that estrogens have a protective effect on the kidney. Furthermore, other studies about the reproductive period showed that a longer reproductive period was associated with a lower risk of chronic diseases such as diabetes, metabolic syndrome, and cardiovascular and cerebrovascular events [17,18,19,40]. Brand et al. [41] showed that the risk of diabetes was 6% lower for each standard deviation in the length of the reproductive period. Indeed, estrogens can help control glucose, and exogenous estrogens are beneficial to insulin secretion and glucose homeostasis [42]. The possible mechanisms include direct effects of estrogens on the pancreas, estrogen-induced increases in pancreatic islet progesterone receptors, estrogen-induced increases in glucocorticoid activity, estrogen-induced increases in growth hormone activity, and estrogen-induced decreases in glucagon sensitivity and secretion. Another study found that the reproductive period was significantly associated with blood lipid levels, insulin sensitivity, and body composition [39]. Since diabetes, metabolic syndrome, and cardiovascular and cerebrovascular events are risk factors for CKD, preventing these conditions will indirectly affect the eGFR. In addition, association between length of reproductive period and risk of decreased eGFR is usually attributed to a shorter or prolonged exposure to endogenous estrogens. Estrogen can regulate the synthesis of growth promoters and inhibitors, inhibiting the proliferation of glomerular mesangial cells [43]. Animal models have shown that glomerular fibrosis could be reduced using estrogen therapy and that the renal injury progression was slower in females than in males [44]. In addition, a 10-year prospective study found that menopausal women with estrogen therapy had better eGFR control than nonusers, suggesting the association between the period of estrogen exposure during which estrogens protect the kidney, leading to a low incidence of decreased eGFR [45]. Although we speculate that this lower risk in chronic diseases is due to estrogen, estrogen therapy in postmenopausal women has been associated with increased risk of dementia and CVD in the study of Women’s Health Initiative (WHI) and the Women’s Health Initiative Memory Study (WHIMS) [46,47]. However, subsequent secondary analyses from the Kronos Early Estrogen Prevention Study (KEEPS) and the Early Versus Late Invention Trial (ELITE) demonstrated that these risks were influenced by the woman’s age and time since menopause, with lower absolute risks and hazard ratios for younger than older women [48,49]. Further studies could investigate the relationship between estrogen therapy and chronic diseases in women with different characteristics.

Marginal effects are popular in some disciplines (e.g., economics) because they often provide a good approximation of the amount of change on the y-axis produced by a 1-unit change on the x-axis. In the present study, the relationship of the change in the incidence of decreased eGFR was investigated by a 1-unit change in MET among groups with different reproductive periods in menopausal women by conducting a marginal effect analysis. To our best knowledge, this is the first study to investigate the marginal effect of performing physical activity on decreased eGFR in menopausal women with different fixed reproductive periods. The meaning of this lies in the following: when a woman enters the stage of menopause, her endogenous estrogens decrease, and her reproductive period becomes fixed.

Women with different reproductive periods may have different optimal amounts of physical activity to lower the risk of developing decreased eGFR. In this study, for women with a reproductive period < 30 years, the risk of developing decreased eGFR would increase with the increment of MET before reaching 1500, and their optimal MET was low (around 100). On the other hand, for women with a reproductive period > 30 years, the risk of developing decreased eGFR would decrease with the increment of MET, and their optimal MET was high (around 6000). Previous studies about performing physical activity in menopausal women found that regular physical activity can help menopausal women control weight, prevent muscle loss, and reduce the risk of diabetes and other diseases [8]. A meta-analysis found that physical activity can help improve the eGFR (by 5.22 mL/min/1.73 m^2^) with short-term exercise (<3 months), but not with 3–6 months of exercise or 6–12 months of exercise in CKD patients, indicating the diverse effects of different modes and amounts of exercise on eGFR [50]. In contrast, a cross-sectional study based on the Chinese population reported that >7.5 MET-hours per week was significantly associated with the risk of decreased eGFR in participants without a history of kidney diseases, aged from 55–65 years, but not in participants younger than 55 or older than 65 years [51]. It is hypothesized that in certain age groups of the Chinese population, light-to-moderate physical exercise increases the risk of renal dysfunction. The present study also showed an increased OR of developing eGFR < 60 mL/minute/1.73 m^2^ within <1000 MET in women with a reproductive period of 20–30 years. As for moderate-to-vigorous intensity activity, the imbalance between the reactive oxygen species (ROS) and antioxidants produced by excessive physical activity leads to increased lipid peroxidation associated with chronic kidney disease [52,53]. Further studies could investigate this issue. The present study might suggest a new way to assess the optimal amount of physical activity for women with different reproductive periods.

This study has several strengths. Firstly, we used a community-based population, allowing our results to be generalizable to other groups. The sample size was large, and the background of the population was diverse. This study included both surgical-induced and naturally menopausal women. The SSACB includes many quality control processes, including double-checking and cross-referencing [33]. Furthermore, this is the first study in China to investigate the relationship between the reproductive period and decreased eGFR and to investigate the marginal effect of physical activity with a fixed reproductive period on decreased eGFR. This study has a prospective cohort design; therefore, the ability to elucidate the causal inference between the reproductive period and eGFR is possible. In addition, in this study, we take hormone therapy into consideration, which can make the results more reliable and more similar to reality.

Still, this study also has limitations. The data on menopause were collected by self-reported questionnaire; therefore, there is a possibility of information and recall biases. Using only single measurements of eGFR and proteinuria in the cross-sectional analyses would also be a limitation. Further evaluations more accurately discerning CKD would be necessary. This study did not include the period of pregnancy and hormone therapy. Further study could include them as covariates, which could make the results more reliable. Furthermore, this study did not consider primary ovarian insufficiency and women who underwent hysterectomy separately; further study could interpret them separately and compare the results with the naturally menopausal women.

## 5. Conclusions

In conclusion, in this community-based study of Chinese menopausal women, the reproductive period was associated with decreased eGFR after adjustment for age, BMI, parental history of chronic kidney disease, age at menarche, pregnancies, hormone replacement therapy, education, smoking, alcohol intake, hypertension, and hyperlipidemia. Women with a longer reproductive period might have a lower risk of developing decreased renal function, and performing physical activity decreased the risk of decreased renal function in women with a longer reproductive period.

## Figures and Tables

**Table 1:** Comparison of baseline characteristics of postmenopausal women with different length reproductive period.

Variables	Total (*n* = 5503)	Length of Reproductive Period (Years)					*p*
		Q1: 18–31 (*n* = 1103)	Q2: 31–33 (*n* = 1105)	Q3: 33–35 (*n* = 1098)	Q4: 35–37 (*n* = 1102)	Q5: 37–45 (*n* = 1095)	
Reproductive period (years)	34.0 (18.0–45.0)	29.0 (18.0–31.0)	32.0 (31.0–33.0)	34.0 (33.0–35.0)	36.0 (35.0–37.0)	39.0 (37.0–45.0)	<0.001
Age (years)	61.0 (36.0–74.0)	63.0 (36.0–74.0)	62.0 (45.0–74.0)	59.0 (38.0–74.0)	60.0 (48.0–74.0)	60.0 (46.0–74.0)	<0.001
Reason of menopause							<0.001
Aging	5183 (94.2%)	915 (83.0%)	1060 (95.9%)	1051 (95.7%)	1077 (97.7%)	1080 (98.6%)	
Unnatural	320 (5.8%)	188 (17.0%)	45 (4.1%)	47 (4.3%)	25 (2.3%)	15 (1.4%)	
Age at menarche (years)	16.0 (13.0–19.0)	17.0 (13.0–19.0)	17.0 (13.0–19.0)	16.0 (13.0–19.0)	16.0 (13.0–19.0)	15.0 (13.0–19.0)	<0.001
Age at menopause (years)	50.0 (37.0–58.0)	45.0 (37.0–50.0)	50.0 (44.0–52.0)	50.0 (46.0–54.0)	52.0 (48.0–56.0)	54.0 (50.0–58.0)	<0.001
Postmenopausal period (years)	10.0 (0.0–36.0)	18.0 (0.0–36.0)	13.0 (0.0–26.0)	9.0 (0.0–25.0)	8.0 (0.0–24.0)	5.0 (0.0–23.0)	<0.001
Pregnancy times							<0.001
0	31 (0.6%)	9 (0.8%)	7 (0.6%)	6 (0.5%)	5 (0.5%)	4 (0.4%)	
1	834 (15.2%)	113 (10.2%)	135 (12.2%)	188 (17.1%)	207 (18.8%)	191 (17.4%)	
2	2304 (41.9%)	471 (42.7%)	452 (40.9%)	453 (41.3%)	481 (43.6%)	447 (40.8%)	
≥3	2334 (42.4%)	510 (46.2%)	511 (46.2%)	451 (41.1%)	409 (37.1%)	453 (41.4%)	
BMI (kg/m^2^)	24.4 (14.6–43.6)	24.5 (15.4–40.7)	24.2 (14.9–42.1)	24.0 (15.6–43.6)	24.4 (15.4–40.8)	24.9 (14.6–39.1)	<0.001
Education level							<0.001
Low	3718 (67.6%)	841 (76.2%)	823 (74.5%)	687 (62.6%)	669 (60.7%)	698 (63.7%)	
Intermediate	1767 (32.1%)	258 (23.4%)	281 (25.4%)	406 (37.0%)	429 (38.9%)	393 (35.9%)	
High	18 (0.3%)	4 (0.4%)	1 (0.1%)	5 (0.5%)	4 (0.4%)	4 (0.4%)	
Smoking							0.29
Never	5496 (99.9%)	1100 (99.7%)	1105 (100.0%)	1098 (100.0%)	1101 (99.9%)	1092 (99.7%)	
Former	1 (<1%)	0 (0.0%)	0 (0.0%)	0 (0.0%)	0 (0.0%)	1 (0.1%)	
Current	6 (0.1%)	3 (0.3%)	0 (0.0%)	0 (0.0%)	1 (0.1%)	2 (0.2%)	
Family history of CKD	54 (1.0%)	10 (0.9%)	12 (1.1%)	15 (1.4%)	8 (0.7%)	9 (0.8%)	0.58
Alcohol intake	34 (0.6%)	5 (0.5%)	9 (0.8%)	2 (0.2%)	9 (0.8%)	9 (0.8%)	0.20
T2DM	1047 (19.0%)	206 (18.7%)	213 (19.3%)	220 (20.0%)	209 (19.0%)	199 (18.2%)	0.85
Hypertension	3268 (61.2%)	663 (62.6%)	656 (61.3%)	648 (60.0%)	630 (58.7%)	671 (63.5%)	0.15
Past hormone replacement therapy	787 (15.2%)	137 (13.2%)	174 (16.8%)	169 (16.3%)	146 (14.1%)	161 (15.5%)	0.12
Laboratory parameters							
BUN (mg/dL)	15.1 (3.4–33.9)	15.1 (5.6–33.9)	15.1 (6.2–33.9)	14.9 (3.4–28.6)	14.9 (7.0–31.1)	15.1 (6.7–33.9)	0.080
Cr (mg/dL)	0.7 (0.1–1.1)	0.7 (0.4–1.0)	0.7 (0.3–1.0)	0.7 (0.4–1.0)	0.7 (0.1–1.1)	0.7 (0.1–1.0)	0.046
HbA1c (%)	5.8 (3.6–14.7)	5.8 (3.8–14.7)	5.8 (3.6–13.0)	5.8 (3.6–12.4)	5.8 (4.0–13.2)	5.8 (3.7–13.0)	0.601
TC (mg/dL)	197.7 (73.3–508.9)	197.7 (88.8–481.4)	198.1 (95.7–350.4)	196.5 (73.3–508.9)	198.1 (87.6–373.6)	197.6 (85.7–426.7)	0.610
HDL-C (mg/dL)	57.0 (14.0–180.2)	57.0 (15.1–180.2)	57.0 (20.2–155.0)	57.8 (20.2–118.2)	56.6 (17.4–113.6)	57.4 (14.0–109.7)	0.562
TG (mg/dL)	127.4 (29.2–1347.0)	129.2 (41.6–1347.0)	125.7 (30.1–1342.0)	124.8 (29.2–994.7)	128.3 (38.1–1110.0)	127.4 (45.1–1296.0)	0.570
LDL-C (mg/dL)	111.6 (0.0–327.0)	110.4 (0.0–327.0)	113.5 (15.4–257.1)	111.2 (3.9–320.8)	111.6 (10.8–279.9)	111.6 (4.2–317.8)	0.630
ALB (g/dL)	5.0 (3.3–6.1)	4.9 (3.3–6.1)	5.0 (4.0–5.8)	5.0 (3.9–6.0)	5.0 (4.0–5.8)	4.9 (3.9–5.8)	0.808
Hb (g/dL)	13.5 (5.8–18.0)	13.4 (5.8–16.2)	13.4 (9.7–16.5)	13.5 (9.9–18.0)	13.5 (11.0–16.2)	13.5 (8.4–15.9)	0.189
UA (mg/dL)	4.6 (1.8–10.8)	4.6 (2.1–10.0)	4.5 (1.8–9.8)	4.5 (1.8–10.8)	4.6 (2.0–9.3)	4.6 (2.3–9.8)	0.209
eGFR (mg/dL)	92.2 (60.1–194.5)	90.7 (60.7–117.3)	91.2 (60.8–130.4)	92.9 (60.3–116.4)	93.2 (60.1–177.0)	92.6 (60.3–194.5)	<0.001
MET	1386 (160–6678)	1386 (160–6678)	1386 (160–6678)	1386 (160–6678)	1440 (160–6678)	1386 (160–6678)	0.164

BMI, body mass index; CKD, chronic kidney disease; T2DM, Type 2 diabetes mellitus; BUN, blood urea nitrogen; Cr, creatinine; HbA1c, glycated hemoglobin; TC, total cholesterol; HDL-C, high-density lipoprotein cholesterol; TG, triglycerides; LDL-C, low-density lipoprotein cholesterol; Hb, hemoglobin; ALB, albumin; UA, uric acid; eGFR, estimated glomerular filtration rate; MET, Metabolic equivalent. Data are expressed as median (range) or *n* (%). *p*-values were calculated using Kruskal–Wallis rank test and Pearson χ2 test.

**Table 2:** ORs (95% CI) for decreased renal function in postmenopausal women with different reproductive period.

	Model 1		Model 2		Model 3	
	OR (95% CI)	*p*	OR (95% CI)	*p*	OR (95% CI)	*p*
Reproductive period						
Q1: 18–31	1.00	-	1.00	-	1.00	-
Q2: 31–33	0.83 [0.56,1.25]	0.375	0.73 [0.47,1.16]	0.187	0.68 [0.43,1.09]	0.109
Q3: 33–35	0.73 [0.46,1.16]	0.185	0.58 [0.35,0.96]	0.035	0.56 [0.34,0.94]	0.027
Q4: 35–37	0.66 [0.41,1.05]	0.082	0.68 [0.41,1.13]	0.139	0.66 [0.40,1.10]	0.113
Q5: 37–45	0.53 [0.30,0.96]	0.036	0.65 [0.39,1.07]	0.091	0.59 [0.35,1.00]	0.049
Per 1-year increase	0.96 [0.93,1.00]	0.030	0.97 [0.94,1.01]	0.144	0.97 [0.93,1.00]	0.071

Data are expressed as odds ratio (95% confidence interval). The odds ratio (OR), 95% confidence interval (CI), and *p*-values were analyzed using logistic regression analysis. Model 1: age; model 2: age, BMI, parental history of chronic kidney disease, age at menarche, pregnancies, hormone replacement therapy; model 3: age, BMI, parental history of chronic kidney disease, age at menarche, pregnancies, hormone replacement therapy, education, smoking, alcohol intake, hypertension, hyperlipidemia, blood urea nitrogen, hemoglobin, albumin, uric acid, and metabolic equivalents.

**Table 3:** The interaction of reproductive period and metabolic equivalent on decreased renal function.

	OR (95% CI)	*p*
Reproductive period		
Q1: 18–31	1.00 [1.00,1.00]	.
Q2: 31–33	0.74 [0.46,1.20]	0.219
Q3: 33–35	0.51 [0.29,0.92]	0.024
Q4: 35–37	0.74 [0.44,1.27]	0.276
Q5: 37–45	0.61 [0.34,1.08]	0.091
MET	1.10 [0.73,1.65]	0.656
Reproductive period × MET		
Q1 × MET	1.00 [1.00,1.00]	.
Q2 × MET	1.22 [0.61,2.42]	0.573
Q3 × MET	1.98 [0.91,4.29]	0.083
Q4 × MET	0.87 [0.42,1.80]	0.712
Q5 × MET	0.43 [0.23,0.81]	0.01

Data are expressed as odds ratio (95% confidence interval). The odds ratio (OR), 95% confidence interval (CI), and *p*-values were analyzed using logistic regression analysis. Adjusted variable: age, body mass index, parental history of chronic kidney disease, age at menarche, pregnancies, hormone replacement therapy, education, smoking, alcohol intake, hypertension, hyperlipidemia, blood urea nitrogen, hemoglobin, albumin, and uric acid.

## Data Availability

All data generated or analyzed during this study is available from the corresponding author upon reasonable request.

## Supplementary Materials

The following are available online at https://www.mdpi.com/article/10.3390/ijerph181910451/s1, Table S1: eGFR categories in menopausal women with different length reproductive period at follow-up.
