# Supplementary material for: Association of the Reproductive Period with Decreased Estimated Glomerular Filtration Rate in Menopausal Women: A Study from the Shanghai Suburban Adult Cohort and Biobank (2016–2020)
Source: Int J Environ Res Public Health. 2021 Oct 5;18(19):10451. doi: 10.3390/ijerph181910451 (PMC8508464; doi:10.3390/ijerph181910451)
Supplement: Supplementary file 1 [file ijerph-18-10451-s001.zip › ijerph-1367092-supplementary.pdf]

International Journal of
Environmental Research and Public Health

MDPI

Supplement Table S1. eGFR categories in menopausal women with different length reproductive period at follow-up.

Variables	Total (N=5503)	Length of reproductive period (years)					P
		Q1: 18-31 (N=1103)	Q2: 31-33 (N=1105)	Q3: 33-35 (N=1098)	Q4: 35-37 (N=1102)	Q5: 37-45 (N=1095)	
**eGFR (mg/dL)**							<0.001
≥90	2620 (47.6%)	464 (42.1%)	467 (42.1%)	573 (52.1%)	563 (51.1%)	553 (50.7%)	
60-89	2691 (48.9%)	574 (52.1%)	602 (54.2%)	499 (45.4%)	507 (46.0%)	509 (46.7%)	
45-59	168 (3.0%)	54 (4.9%)	36 (3.2%)	26 (2.4%)	28 (2.5%)	24 (2.2%)	
30-44	23 (0.4%)	8 (0.7%)	5 (0.5%)	2 (0.2%)	3 (0.3%)	5 (0.5%)	
15-29	1 (2%)	1 (0.1%)	0 (0.0%)	0 (0.0%)	0 (0.0%)	0 (0.0%)	

eGFR, estimated glomerular filtration rate. Data were expressed as median (range) or n(%). P-values were calculated using Kruskal, Wallis rank test, Pearson $\chi 2$ test.
